# The STAR trial protocol: a randomised multi-stage phase II/III study of Sunitinib comparing temporary cessation with allowing continuation, at the time of maximal radiological response, in the first-line treatment of locally advanced/metastatic Renal Cancer

**DOI:** 10.1186/1471-2407-12-598

**Published:** 2012-12-14

**Authors:** Fiona J Collinson, Walter M Gregory, Chris McCabe, Helen Howard, Catherine Lowe, DrBarbara Potrata, Sandy Tubeuf, Pat Hanlon, Lucy McParland, T Wah, Peter J Selby, Jenny Hewison, Julia Brown, Janet Brown

**Affiliations:** 1 Clinical Trials Research Unit, University of Leeds, Leeds, LS2 9JT, UK; 2 Academic Unit of Health Economics, Leeds Institute of Health Sciences, Charles Thackrah Building, University of Leeds, 101 Clarendon Road, Leeds, LS2 9LJ, UK; 3 Charles Thackrah Building, Leeds Institute of Health Sciences, University of Leeds, Leeds, LS2 9JT, UK; 4 Academic Unit of Health Economics, Leeds Institute of Health Sciences, Charles Thackrah Building, University of Leeds, 101 Clarendon Road, Leeds, LS2 9LJ, UK; 5 Patient Representative National Cancer Research Institute (NCRI) Renal Cancer Clinical Studies Group, Leeds, UK; 6 Department of Radiology St James’s University Hospital, Leeds, LS9 7TF, UK; 7 Cancer Research Building, St James’s University Hospital, Leeds, LS9 7TF, UK; 8 Charles Thackrah Building, Leeds Institute of Health Sciences, University of Leeds, Leeds, LS2 9TF, UK; 9 Cancer Research UK Experimental Centres at Leeds and Sheffield, Leeds, LS2 9TF, UK

**Keywords:** Renal cancer, Sunitinib, Intermittent treatment, Quality of life, Quality adjusted life years, Health economics

## Abstract

**Background:**

Over recent years a number of novel therapies have shown promise in advanced renal cell carcinoma (RCC). Internationally the standard of care of first-line therapy is sunitinib™, after a clear survival benefit was demonstrated over interferon-α. Convention dictates that sunitinib is continued until evidence of disease progression, assuming tolerability, although there is no evidence that this approach is superior to intermittent periods of treatment. The purpose of the STAR trial is to compare the standard treatment strategy (conventional continuation strategy, CCS) with a novel drug free interval strategy (DFIS) which includes planned treatment breaks.

**Methods/Design:**

The STAR trial is an NIHR HTA-funded UK pragmatic randomised phase II/III clinical trial in the first-line treatment of advanced RCC. Participants will be randomised (1:1) to either a sunitinib CCS or a DFIS. The overall aim of the trial is to determine whether a DFIS is non-inferior, in terms of 2-year overall survival (OS) and quality adjusted life years (QALY) (averaged over treatment and follow up), compared to a CCS. The QALY primary endpoint was selected to assess whether any detriment in terms of OS could be balanced with improvements in quality of life (QoL). This is a complex trial with a number of design challenges, and to address these issues a feasibility stage is incorporated into the trial design. Predetermined recruitment (stage A) and efficacy (stage B) intermediary endpoints must be met to allow continuation to the overall phase III trial (stage C). An integral qualitative patient preference and understanding study will occur alongside the feasibility stage to investigate patients’ feelings regarding participation or non-participation in the trial.

**Discussion:**

The optimal duration of continuing sunitinib in advanced RCC is unknown. Novel targeted therapies do not always have the same constraints to treatment duration as standard chemotherapeutic agents and currently there are no randomised data comparing different treatment durations. Incorporating planned treatment breaks has the potential to improve QoL and cost effectiveness, hopefully without significant detriment on OS, as has been demonstrated in other cancer types with other treatments.

**Trial Registration:**

Controlled-trials.com ISRCTN 06473203

## Background

### Renal cell carcinoma

Renal cell carcinoma (RCC) constitutes only 3% of adult malignancies, but is the sixth leading cause of cancer-related death due to the lack of effective therapy for locally advanced and metastatic disease.

The strategy of targeting angiogenic pathways has produced positive results in advanced RCC. Tyrosine kinase inhibitors (TKIs), e.g. sunitinib and sorafenib, and monoclonal antibodies, e.g. bevacizumab with IFNα, have produced improvements in terms of progression free survival (PFS) and overall survival (OS). Sunitinib (Sutent™) selectively targets multiple protein receptor tyrosine kinases including vascular endothelial growth factor receptor and platelet-derived growth factor receptor. This inhibits tumoural delivery of blood and nutrients required for growth, which ultimately leads to cancer cell death. Sunitinib also has a direct inhibitory effect on tumour cells [1].

The landmark sunitinib trial in RCC was a double-blind randomised controlled trial of 750 patients (ECOG performance status 0 or 1) with metastatic RCC which compared sunitinib to IFNα as first-line therapy [2]. The primary endpoint was PFS and the trial was unblinded after a second interim analysis demonstrated significant benefit in patients treated with sunitinib. Updated results were published in 2009 and demonstrated a median PFS of 11 months with sunitinib and 5 months with IFNα in the intention-to-treat population (p<0.001) [3]. The OS was 26.4 months with sunitinib and 21.8 months with IFNα (HR 0.821; 95% confidence intervals [CI]=0.673-1.001, p=0.051), however this difference is likely to be an underestimate due to significant crossover from IFNα to sunitinib after unblinding. Sunitinib was also associated with improved response rates over IFNα (47% vs 12%).

Sunitinib was approved by NICE in 2009 for UK use in the first-line treatment of advanced RCC in patients with a good performance status (ECOG 0 or 1) until evidence of disease progression or unacceptable toxicity [4]. This was after reappraisal under the specific ‘end-of-life’ criteria as it had not previously been approved under standard NICE decision-making criteria.

Sunitinib is associated with a significant side effect burden. The landmark first-line trial reported that 19% of patients discontinued sunitinib due to adverse events (AEs) and 50% of patients required a dose reduction [3]. In the sunitinib open access program 8% of patients discontinued drug due to serious adverse events (SAEs) and a further 30% had dose reductions due to toxicity [5]. Commonly reported AEs included hypertension (12%), fatigue (11%), diarrhoea (9%) and hand-foot syndrome (9%) [3]. The longer-term impact of sunitinib-associated toxicities are also recognised to be increasingly important as patients are living longer [6].

### Intermittent anti-cancer treatment strategies

There is increased interest in drug-free interval strategies (DFIS) in oncology with evidence that these approaches are associated with reduced toxicity and improved quality of life (QoL), without significantly compromising survival benefits. This approach is most studied in colorectal cancer (CRC), where there is evidence that planned treatment breaks (a DFIS) can be utilised with no, or a minimal, OS deficit, but with an associated advantage in terms of improved QoL [7-10].

OPTIMOX1 compared 6 cycles of FOLFOX7 (three weekly bolus oxaliplatin, 5-fluorouracil [5FU] and folinic acid [FA]) followed by 5FU/FA alone (for up to 24 weeks) before re-introduction of oxaliplatin, to FOLFOX4 (two weekly bolus oxaliplatin, 5FU and FA) until progression, in 623 patients with metastatic CRC [9]. The results from this trial demonstrated that duration of disease control was similar between both arms (10.6 and 9.0 months respectively), as were PFS and OS. Importantly almost 60% of patients on the intermittent arm did not have oxaliplatin reintroduced at the appropriate timepoint, but those that did tended to have a better outcome [11]. Not reintroducing drugs as per protocol recommendation has been a significant issue in a number of the trials investigating a DFIS. The subsequent OPTIMOX2 trial compared complete breaks from chemotherapy with continued 5FU/FA until progression, but unfortunately was stopped prematurely after recruitment of only 216 of the planned 600 patients as the standard arm became obsolete due to the availability and use of bevacizumab. Preliminary results demonstrated improved duration of disease control (13.1 vs 9.2 months) and OS (23.8 vs 19.5 months) in the continuous arm, however there have been criticisms levied at the trial design, in particular the primary endpoint of duration of disease control, as treatment for progressive disease was not mandated until the disease reached baseline size, not at the time of initial progression compared to best response as is standard practice. The statistical plan was also not adapted to account for the reduced sample size from 600 to 216. This prevents definitive conclusions being drawn from the results [7].

In the UK this concept of planned treatment breaks was further investigated with the large randomised COIN trial [10]. In this study 1639 patients receiving oxaliplatin plus fluoropyrimidine-based chemotherapy were randomly assigned (1:1) to continuous chemotherapy until progression (arm A) or intermitted chemotherapy (arm C). In arm C, patients at 12 weeks who were responding, or who had stable disease, stopped chemotherapy until evidence of clinical or radiological disease progression. Whilst the results were unable to confirm non-inferiority of intermittent chemotherapy (the continuous arm demonstrated an improvement in OS of 1.4 months, HR 1.084, 80%CI 1.008-1.165), it was concluded that intermittent treatment is a reasonable option in fully informed patients due to the reductions in cumulative toxicities and improvements in QoL, traded off with minimal detriment in terms of OS. Treating patients with CRC with pre-planned chemotherapy breaks remains standard practice in many centres internationally.

A number of smaller studies provide further support for a sunitinib DFIS. One study included 23 patients with metastatic RCC who had previously responded to sunitinib, and then received alternative therapies at progression (median duration 6.7 months) who were then re-challenged with sunitinib at the time of further progression. In this cohort a second sunitinib-related PFS of 7.2 months (median) was achieved [12]. Importantly no additional or increased toxicities were observed on re-challenge. Another small retrospective study reported the effects of stopping sunitinib therapy in 11 patients who had had a complete response, with or without surgical metastectomy following response to sunitinib [13]. At median follow up of 8.5 months, disease had recurred in 5 patients, but in all cases re-introduction of sunitinib was effective in regaining disease control.

There is also one randomised phase II study in which 202 patients with metastatic RCC were treated with sorafenib (an alternative TKI). After 12 weeks of sorafenib, the 65 patients with stable disease (SD) were randomly assigned to continue sorafenib (n=32) or placebo (n=33). At 24 weeks 50% of patients continuing sorafenib were progression-free compared with 18% of placebo-treated patients (p=0.0077), however, when sorafenib was re-administered in 28 of the placebo-treated patients with disease progression, further progression was delayed for a median of 24 weeks. The researchers concluded that intermittent treatment with sorafenib resulted in comparable median summative PFS as for those patients receiving continuous sorafenib [14]. This suggests that patients were not disadvantaged from a brief period of placebo treatment, providing further ethical support for the design of the STAR trial.

At present there is no clearly defined optimal treatment strategy for many targeted therapies, and research in this field is crucial both for patients in terms of QoL and for the National Health Service (NHS) in terms of cost-effectiveness. Regarding sunitinib in advanced RCC, evidence for the cost-effectiveness is currently poor and standard decision criteria did not support its implementation in the NHS. The STAR trial will address the need to gather robust evidence on the costs, QoL and clinical outcomes of sunitinib using both a DFIS and a conventional continuation strategy (CCS). If successful, the design may be applicable to other drugs across a wide range of diseases.

## Methods/design

### Trial aims and objectives

This trial will determine whether by utilising a sunitinib DFIS in advanced RCC, survival benefits can be maintained, whilst other important outcomes such as QoL and cost-effectiveness, can be improved, compared to utilising a sunitinib CCS. The STAR trial is a three-stage phase II/III trial with stages A and B incorporated into the phase II trial component and Stage C incorporated into the phase III trial (which includes participants from all 3 stages).

#### Overall primary objectives

• To determine whether a sunitinib DFIS is non-inferior in terms of 2-year OS and quality-adjusted life years (QALYs) (averaged over trial recruitment and follow-up) compared to a sunitinib CCS, in patients with locally advanced and/or metastatic clear cell RCC.

Oncological treatments for patients with incurable disease require assessment using standard measures of efficacy, such as survival. It is however recognised that other measures must also be taken into consideration including QoL and cost, particularly in the context of the economic constraints of the NHS. When seeking approval from NICE for a new treatment, all of these outcomes are considered. A composite co-primary endpoint (QALY averaged over trial recruitment and follow up) was selected as a primary outcome measure to more accurately assess whether any detriment in survival could be balanced with improvements in QoL and cost-effectiveness. Limitations of the previously reported QoL data relating to advanced RCC means significant uncertainty remains about the profile, timing and magnitude of the QoL impact of sunitinib treatment, hence OS has been retained as a co-primary endpoint.

#### Stage specific primary objectives

*Stage A:* To establish the feasibility of performing the phase III trial in terms of average recruitment between months 10–21 (inclusive). This is to ensure that sufficient participants can be recruited to enable its completion in a timely manner, assuming continuation to stage C.

*Stage B***:** To provide initial efficacy data by comparing time to strategy failure^a^ (TSF) in both arms and test for non-inferiority between the approaches to assess comparability.

*Stage C:* 2 year OS and QALY averaged over study recruitment and follow up

#### Secondary objectives

The secondary objectives are to evaluate how utilisation of DFIS compared to utilisation of a CCS impacts on: 

• Summative Progression Free Interval (SPFI)^b^

• Time to Strategy Failure (TSF)

• Toxicity (CTCAE v.4.0)

• QoL

• Cost-effectiveness

• Progression free survival (PFS)

### Trial population

The STAR trial population is planned to be inclusive of all patients who would be eligible to receive sunitinib in the UK, in accordance with current NICE guidance and its marketing licence. Suitable patients will be male or female, aged ≥ 18 years old, with a histologically confirmed clear cell inoperable locally advanced or metastatic RCC, having had no prior systemic therapy for advanced disease. Patients will be ECOG performance status 0–1 and be required to have uni-dimensionally measurable disease as per RECIST criteria. Patients will be required to have appropriate haematological and biochemical parameters and to provide written informed consent.

Allowable situations include patients with either primary renal cancer in-situ or having undergone a previous nephrectomy, patients with previously diagnosed brain metastases treated with complete surgical resection or gamma knife therapy with no subsequent evidence of progression (patients treated with whole brain radiotherapy are not eligible) and patients previously treated on the SORCE sorafenib adjuvant trial, providing they did not receive sorafenib. Patients are also eligible to participate in the STAR trial having had previous radiotherapy and/or previous/ongoing bisphosphonates for the treatment of symptomatic bony metastasis.

Patients are not eligible to participate in the STAR trial with pulmonary or mediastinal disease causing obstruction and/or haemoptysis, if they have an estimated life expectancy of <6 months, if they have any known contraindications to sunitinib (including hypersensitivity) or have had any previous treatment with sunitinib or other tyrosine kinase inhibitor (including in the adjuvant setting). Patients will also be ineligible to participate if they are taking any concomitant medications known to significantly affect the activity or pharmacokinetics of sunitinib or if they have poorly controlled hypertension despite maximal medical therapy. Patients with any other serious medical or psychiatric condition, which in the opinion of the investigator could affect participation in the STAR trial will also be excluded.

### Trial design

**Figure 1:**
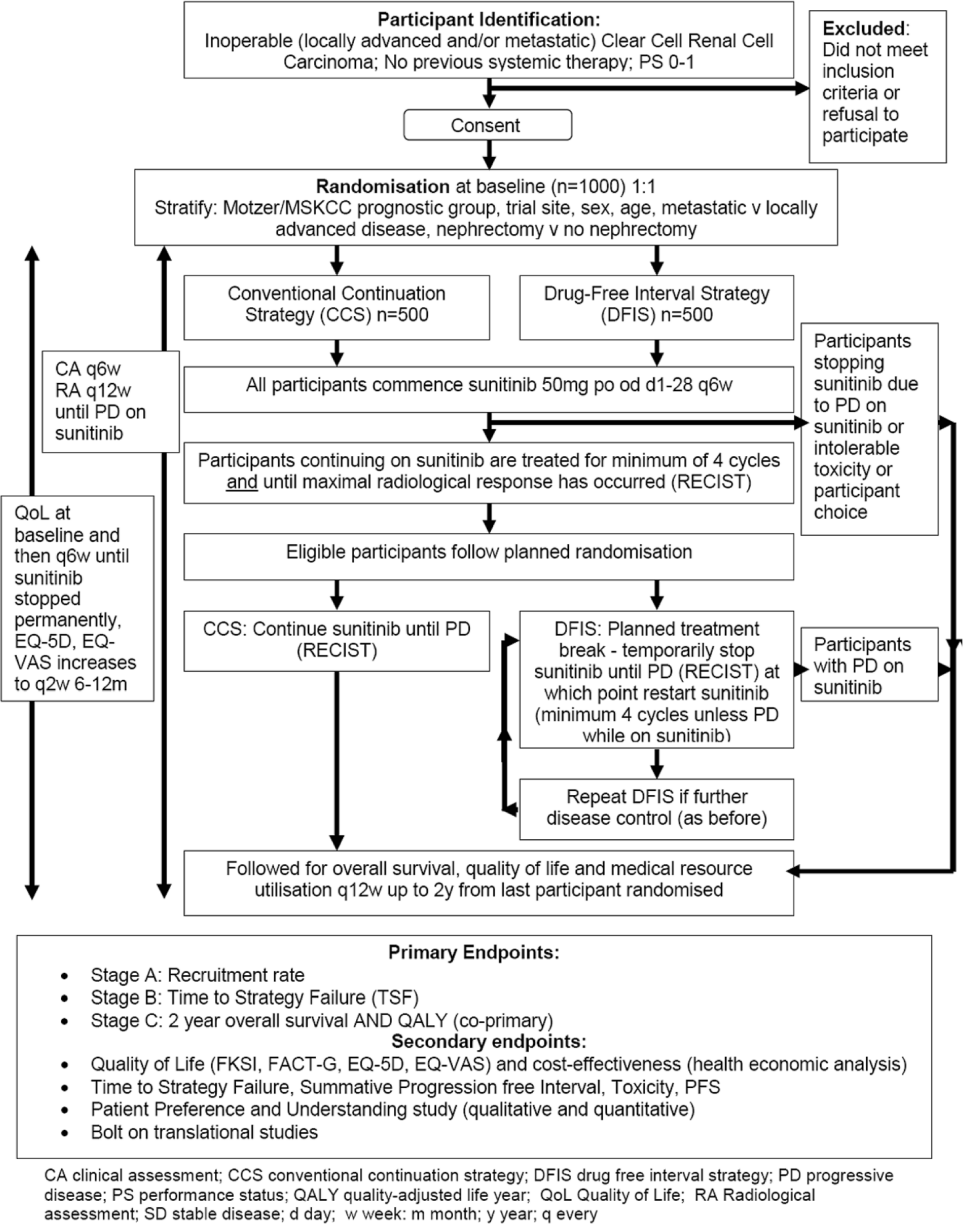
Summary of the STAR trial.

This is a pragmatic UK, multi-centre two-arm, randomised (1:1) controlled, multi-stage, open-label phase II/III trial in patients with inoperable locally advanced or metastatic clear cell renal cell cancer to evaluate the use of a sunitinib DFIS compared to the sunitinib CCS (see Figure 1). It is a particularly appropriate trial in the UK due to the need to develop treatment strategies that will allow patients access to treatments, which are delivered in the most beneficial and cost-effective way in the NHS. There are design challenges relating to this trial and a multi-stage approach has been utilised to optimise the effectiveness of the design in terms of time and sample size. The trial must meet pre-determined intermediary endpoints in stages A and stage B for recruitment to continue into stage C. Achievement of stage-specific endpoints will be assessed by an independent data monitoring and ethics committee (DMEC). 

### Sample size

In total, for all 3 stages, 1000 patients will be required (allowing for a 10% drop-out), 210 in stages A/B (phase II), and a further 790 in stage C (phase III). Stages A/B will be performed in 13 UK sites, and assuming continuation to stage C, then the trial will be opened up to a further 26 sites. The sample size is based on the co-primary endpoints, however it will also be adequate to obtain meaningful conclusions regarding the key secondary endpoints e.g. QoL.

A computer-generated minimisation programme that incorporates a random element will be used to ensure that treatment groups are well balanced for the following factors: Motzer prognostic group [15] (favourable risk (0 factors) vs. intermediate risk (1–2 factors) vs poor risk (≥ 3 factors)), trial site, sex, age (< 60 years vs. ≥ 60 years), disease status at randomisation (locally advanced vs. metastatic) and whether or not the patient has had a previous nephrectomy.

### Blinding

Blinding of the study was considered, acknowledging that this would be preferable scientifically, particularly in respect of patient reported outcomes. However, due to the well-recognised toxicity profile of sunitinib, and the costs involved, it was decided not to blind this trial. Accurate radiological evaluations will be fundamental to the stage B endpoint (TSF), and for this reason all radiological evaluations in the initial two stages (A and B) will be performed centrally. Due to the need for the radiologist to be certain which scan they are comparing subsequent scans to (the baseline scan will change in the DFIS arm) it was not possible to maintain the blind of the central radiologist. The central radiology report, not the local report, will be used to determine whether sunitinib is stopped, continued or recommenced (depending on individual participant arm and current situation).

### Randomisation timing

Consideration was given to the optimal timing for randomisation, either prior to receiving any treatment (baseline), or just prior to participants taking up their randomisation strategy (DFIS/CCS) (this will be at 6 months for most patients). Randomisation was decided to be performed at baseline and participants’ feelings regarding this will be further explored in a qualitative participant preference and understanding study. There is supportive data demonstrating higher patient uptake of allocated arm if randomisation occurred at baseline from a colorectal trial [16] and a lung cancer trial [17]. This decision was also reinforced from a number of consultations with patients taking sunitinib regarding their randomisation timing preferences.

### Recruitment

Participant recruitment is planned over 21 months in stages A/B, and then to seamlessly continue for a further 33 months assuming continuation to stage C, with a number of other centres opening to recruitment in stage C. All participants will be followed up until 2 years after the last participant is randomised. The duration of the trial is expected to be 84 months including set up, recruitment, follow-up and analysis. Recruitment commenced in January 2012.

### Baseline investigations

Baseline investigations are planned to be in line with standard UK practice for patients receiving sunitinib and, apart from a pregnancy test in appropriate female participants, there are no other trial-specific screening investigations.

### Intervention

Participants in both arms of the trial will receive sunitinib which will be administered orally at 50 mg once daily on days 1 to 28 of the standard 42 day cycle. Daily doses may be modified for toxicity to 37.5 mg and 25 mg, in line with standard clinical practice.

After completion of at least 4 cycles of sunitinib and maximal radiological response, participants will take up their randomisation arm. Participants randomised to the control arm will continue sunitinib according to the CCS i.e. continuing treatment until evidence of disease progression or toxicity precluding continuation. Participants randomised to the experimental arm will continue according to the DFIS i.e. they will temporarily stop sunitinib (a planned treatment break). Participants will remain off treatment until radiologically-confirmed evidence of disease progression. At this time sunitinib will be restarted following the same scheduling and dose as before. In participants who regain further disease control, after completion of a minimum of 4 cycles of sunitinib and maximal radiological response, further planned treatment breaks will be implemented.

All participants will continue with their allocated sunitinib treatment strategy as per protocol (with dose reductions as required) until radiologically-confirmed disease progression occurs whilst taking sunitinib, unacceptable toxicity or participant choice to stop per protocol treatment

### Follow-up

The planned duration of the trial follow-up is until 2 years after the last participant is randomised. During continuation of protocol-defined treatment (sunitinib as per CCS or DFIS) six-weekly clinical assessments and twelve-weekly radiological assessments will occur, in line with standard practice. Where protocol-defined treatment has permanently stopped, details of any subsequent treatment received for renal cancer and the participant’s status will be collected at six months and then on an annual basis.

### Data collection and management

All protocol-required information will be entered onto paper case report forms (CRFs) at each site. Initial participant details will be collected on a baseline CRF, and an additional CRF will be completed at each clinical visit, including details of toxicities relating to sunitinib. Participants will also complete several QoL questionnaires (EQ-5D^TM^/EQ-VAS, FACT-G and FKSI) and a medical resource utilisation (MRU) questionnaire at each visit. During months 6–12 of a participant being on the trial they will also be asked to complete two-weekly EQ-5D^TM^/EQ-VAS questionnaires at home. Text and email reminders will be offered to assist with compliance. Overall data and trial management will be provided by the Clinical Trials Research Unit (CTRU) (University of Leeds, UK) where received data will be monitored for quality and completeness. Any missing data will be pursued until it is received, confirmed as not available or the trial is at analysis.

The independent DMEC will review the safety and ethics of the trial. Detailed un-blinded reports will be prepared by the CTRU for the DMEC at approximately yearly intervals. These reports will include summaries of recruitment, toxicity (rates of occurrences of AEs, SAEs, SARs and SUSARs) progression, strategy failure events and overall survival by treatment group. A formal interim analysis will be reported to the DMEC after the end of stages A and B (21 months after the start of recruitment). A report will be prepared and presented to the DMEC on the interim recruitment rates and early efficacy data (TSF).

### Quality of life

Quality of life is a major consideration in care of people with RCC and is a key component of this trial, which aims to explore the impact on QoL of a DFIS strategy compared to a CCS. The use of averaged QALY as a co-primary endpoint of the trial exemplifies the importance of this. QoL will be assessed with the following questionnaires: FACT-G (28 items in four domains) [18], the FKSI-15 (15 items) [19] and the EuroQol instrument (including EQ-5D^TM^ descriptive system and the EQ visual analogue scale, EQ-VAS) [20].

Extensive QoL data were collected as part of the pivotal sunitinib trial programme [21,22], including both generic measures (FACT-G, EQ-5D^TM^/EQ-VAS) and disease-specific measures (FKSI-15 and FKSI-DRS subscale), but the reporting of the EQ-5D^TM^/EQ-VAS data in the publication was restricted to baseline mean and standard deviations (sd) and modelled average for all follow-up. In addition, QoL data from patients who stopped treatment were not included in the analysis. As a result these data are of limited use for estimating the QALY gains from a sunitinib DFIS strategy in the NHS. A small Japanese trial [23] reported baseline and follow-up EQ-VAS data for day 1 and day 28 of each treatment cycle. These data, plotted graphically, show a sawtooth pattern of QoL whilst taking sunitinib, with highest measures on day 1 and lowest measures on day 28, consistent with clinical experience. The authors report that the same pattern was seen in the EQ-5D^TM^ data, providing reassurance that both these instruments will be sensitive to identify the hypothesised benefit. This intra-cycle variability necessitates frequent QoL measures to accurately capture the information required and hence to maximise the opportunity to identify any QoL/QALY differences between the arms. This drives the timings of QoL data collection.

Information will be collected at clinic visits at baseline, and day 1 of sunitinib cycle 1, 2, 3 and 4. Administration in clinic will enable patients to be supported in their completion, if required, prior to initiation of postal questionnaires. After this timepoint EQ-5D^TM^/EQ-VAS information (a simple 2 page assessment) will be completed every 2 weeks by patients at home, over 18 weeks. At clinical assessment visits the FSKI and FACT-G will continue to be completed 6 weekly. It is the period when CCS arm patients continue treatment and most patients on the DFIS arm stop treatment (i.e. after 6 months), when QoL differences are predicted to be greatest and hence this is when QoL data collection is most intensive. After the intensive collection has finished, collection of all information (including EQ-5D/VAS) will revert to 6 weekly in clinic.

### Health economics

The relative cost-effectiveness of a sunitinib DFIS compared to a sunitinib CCS is another important endpoint in the STAR trial. Treatment strategies that reduce the total quantity of sunitinib administered whilst maintaining total health gain are likely to be significantly more cost-effective than the current standard treatment strategy, thus supporting the DFIS model. The potential QoL gains along with the potential reduced rates of adverse events due to treatment interruption make the use of a DFIS strategy worthy of additional investigation as a cost-saving strategy.

To enable a comprehensive cost-effectiveness analysis, information will be collected on community, primary, secondary and tertiary health care utilisation and third sector care of participants. This will be collected via MRU questionnaires and CRFs at each clinical review. The economic evaluation will consider both the NHS and Personal Social Services (PSS) perspective and a societal perspective. The latter will include out-of-pocket expenses, and the productivity costs to the patients and formal and informal care providers. The analysis will estimate the expected incremental cost per QALY of a sunitinib DFIS compared to a sunitinib CCS.

The extensive QoL data collected in this trial represents a unique source of information valuing health at every point of time. Using each follow-up collection point, the distribution QALY gains will be precisely plotted over time and compared between the two arms (co-primary endpoint of the overall trial). There is uncertainty regarding the utilities of different health states in advanced RCC; i.e. the value of ‘Q’ in the QALY, for example NICE report a range for utility values 0.6 to 0.8 for stable/progression free disease states [4,24]. The results of this trial will answer questions about the risks and benefits of structured treatment interruption (DFIS) and will hopefully allow robust conclusions to be drawn. The uncertainty around the appropriate utilities for specific health states will be examined within a probabilistic sensitivity analysis and using scenario analyses where alternative EQ-5D algorithms are used to attach utilities to specific health states. An interim analysis between stages A/B and C of the QALY data collected will be used to ensure the assumptions used to power the trial on a QALY endpoint are correct. The decision analytic cost effectiveness model will also be used to estimate a value of information analysis (VoI) [25] and provide a methodological framework that explicitly considers the uncertainty surrounding the decision of a health care system to adopt a new technology using the expected value of perfect information (EVPI).

### Statistical methods and analysis

Statistical analysis will be the responsibility of the STAR CTRU Trial Statistician. A full statistical analysis plan will be written before any formal analyses are undertaken.

All non-inferiority analyses will be conducted on both the ITT and per protocol analysis populations. For the superiority endpoints the ITT analysis will be given primacy, however for the non-inferiority endpoints equal weighting will be given to both the ITT analysis and the per-protocol analysis, as the ITT is likely to be the least conservative approach when testing for non-inferiority. An overall two-sided 5% significance level will be used for all superiority endpoint comparisons, and a one-sided 2.5% significance level will be used for all non-inferiority endpoints.

A formal interim analysis will take place after stages A and B (at 21 months after the start of recruitment). A feasibility and efficacy stopping rule have been specified (as detailed below) to ensure the trial is stopped as early as possible in the case of insufficient recruitment (Stage A) or insufficient efficacy (Stage B) in the DFIS arm. The DMEC in light of this interim data will make their recommendations to the Trial Steering Committee who will in turn decide whether the trial can continue to Stage C.

#### Stage A

An essential part of any trial is ensuring that recruitment targets are met. This is formalised in the STAR trial, to ensure that the results can be delivered to time and target. To do this the recruitment per month during months 10–21 (inclusive) of recruitment will be determined; not utilising the initial 9 months which are allowed to enable site set up and familiarity with the trial. To continue the trial, a specific recruitment rate must be attained. The 95% CI for a recruitment rate of one patient per centre per month, with 13 centres for 12 months, is .85-1.15. Therefore a minimum of 0.85 patients per centre open and recruiting per month would be required i.e. 133 patients recruited over the 12 month period, assuming all 13 centres opened and commenced recruitment in a timely manner.

#### Stage B

An interim efficacy endpoint has been included to further ensure the appropriateness of extending recruitment and continuing the trial to stage C. PFS is not an appropriate comparator endpoint in the trial as, due to planned treatment breaks, the initial PFS is likely to be shorter in the DFIS arm compared to the CCS arm. TSF will be analysed (targeted to look at differences in 15 months strategy failure rates) in both arms and non-inferiority will be required to be demonstrated between the arms for the trial to continue to stage C. The TSF in the DFIS arm must be less than 15% worse than in the CCS arm (strategy failure assumed to be 80% at 15 months). Assuming 21 months of accrual and immediate analysis, 80% power, and assuming proportional hazards, this would require 67 events and a population of 112 patients (56 in each arm; approximately 53% of the 210 in total that will be randomised) who take up their randomisation at 6 months.

During the formal interim analysis, an analysis of the utility data so far obtained will also be performed to revise the estimates of the power to detect the composite QALY endpoint and to evaluate possible refinements in the trial design as a result.

#### Stage C

There are two co-primary outcome measures: OS and averaged QALY. The two null hypotheses are that DFIS is not inferior to CCS in terms of OS and QALYs. To calculate a sample size for the primary OS endpoint, a difference of ≤7.5% in OS at two years between the two arms has been assumed to be an acceptable non-inferiority margin (equivalent to a hazard ratio of 0.806). To demonstrate this non-inferiority with 80% power will require the recruitment of approximately 1000 patients (allowing for 10% of patients being lost to follow-up). To calculate a sample size for the primary QALY endpoint a difference of ≤10% in mean QALYs between the two arms has been assumed to be an acceptable non-inferiority margin. While it is hoped that survival will be equivalent between the two arms, a slightly poorer survival in the DFIS arm would be acceptable if offset by a significant QoL gain in these patients, this will be captured through the QALY measure. With a hazard ratio of 0.9 in favour of CCS and 1000 patients recruited, simulations give a power of 84% to show non-inferiority in the QALY endpoint. Both non-inferiority sample sizes are calculated assuming a 1-sided 97.5% confidence interval, as described by Kay [26], and assuming patients are recruited in total over 4.5 years, with a further follow-up of 2 years before evaluating the data.

For Stage B TSF and Stage C OS endpoints, the primary analysis will comprise Kaplan-Meier curves including those adjusted for the minimisation factors [27], giving 95% CIs for the TSF and 2-year survival difference. A a Cox proportional hazards model analysis adjusting for minimisation factors and associated hazard ratios plus 95% CIs for the DFIS vs. CCS comparisons will be pesented, as appropriate for the non-inferiority analysis. The analysis of primacy is the Cox model.

For the QALY endpoint mean differences in QALYs, calculated via the EQ-5D, between the arms, with 95% confidence limits, will be calculated. Multivariate linear regression will be used to adjust for minimisation factors. If the data are not normally distributed, transformations to normality will be investigated. If no such transformations can be found we will analyse the difference between median QALYs and we will calculate confidence intervals for the differences between these medians as described by Campbell and Gardner [28].

### Trial organisation and administration

The STAR trial is funded by the NIHR Health Technology Assessment programme (Grant ref 09/91/21). The trial is sponsored by the University of Leeds and was developed by the STAR trial management group and medical advisory group, with the support of the UK NCRI Renal Clinical Studies Group. The trial is registered (ISRCTN06473203 EudraCT number 2011-001098-16).

Trial supervision will be established according to the principles of Good Clinical Practice (GCP) and in line with the relevant Research Governance Framework within the UK and through adherence with CTRU standard operating procedures (SOP). The trial will be performed in accordance with the recommendations guiding physicians in biomedical research involving human subjects adopted by the 18th World Medical Assembly, Helsinki, Finland, 1964, amended at the 52nd World Medical Association General Assembly, Edinburgh, Scotland, October 2000. Ethical approval in the UK has been obtained through the National Research Ethics Service (ref 11/NW/0246).

A core Internal Project Team, Trial Management Group (TMG), a Trial Steering Committee (TSC) and DMEC will be established. The independent DMEC will be appointed to review the safety and ethics of the trial, alongside trial progress and the overall direction as overseen by the TSC. Interim reports will be presented to the DMEC, in strict confidence, at, at least, yearly intervals. This committee, in light of the interim data, and of any advice or evidence they wish to request, will advise the TSC if there is proof beyond reasonable doubt that one treatment strategy is better.

### Ancilliary studies

#### Patient preference and understanding study

There is an integral qualitative substudy in two parts which aims to investigate patient’s reasons for entering or declining entry into the STAR trial. The reasons why, and under what conditions, patients participate in clinical trials is an under-researched and poorly understood area, especially in clinical trials in advanced cancer. Nevertheless, the reasons for non-participation are frequently related to how the healthcare professionals involved present the design and objectives of the clinical trial to the patient, and how the patient assimilates this information. It is therefore of importance to understand how patients accept the information about potential participation in a particular clinical trial and what their experiences are of information provision relating to the trial. The perspectives of patients as potential users have become an essential part of determining clinical utility of drugs and/or procedures yet patients’ perspectives on being involved in clinical trials are a severely under-researched area.

It is anticipated that this trial will be complex to discuss with patients, who may have reservations and strong feelings about stopping a treatment that they know to be working. There is only limited guidance from the literature regarding how optimally to present this trial to patients as evidence available will be either too generic, or too specific for the individual trial investigated.

This substudy aims to conduct qualitative semi-structured interviews with up to 50 patients from three clinical research sites with different catchment patterns to increase the diversity of the sample. Twenty-five patients who have declined to participate in the STAR trial and 25 STAR participants randomised to the DFIS arm will be included. The patients who have declined participation will be interviewed to investigate their reasons for declining trial entry. The DFIS participants will be interviewed to investigate their feelings about stopping sunitinib, but also the reasons which led them to initially participate. All interviews will be audio-recorded and professionally transcribed verbatim. The analysis will adopt thematic approach.

Information gained will be utilised to optimise patient information provided relating to the study (audiovisual and PIS) and to inform clinical staff regarding preferred methods of describing the study, which ideally will enable recruitment to be maximised in stage C of the study. Other studies (e.g. the ProtecT study which randomised patients with localised prostate cancer to surgery, radiotherapy or active monitoring) have confirmed the value of such approaches in improving recruitment and taking up of randomisation [29].

#### Translational studies

Translational research studies are planned to investigate blood and/or tissue markers of sunitinib response and toxicity, and imaging markers to identify earlier evidence of disease response or progression. Imaging sub-studies include an optional CT study and an optional MRI study.

## Discussion

This has been a challenging trial to design, however the design is applicable to many other treatment strategies using different drugs and in different disease areas. Non-standard endpoints were required due to the intermittent nature of the DFIS; as PFS was not an appropriate key intermediary endpoint, TSF was defined and selected. Similar composite endpoints have been used in the previous intermittent chemotherapy CRC trials, and equivalent endpoints in intermittent therapy trials of anti-retrovirals in the treatment of HIV and of antipsychotics in Alzheimer’s disease.

Another issue during design was the use of pazopanib (Votrient^TM^, GSK), another TKI which works via the same mechanism as sunitinib. In the UK it has recently been recommended by NICE as an alternative first-line treatment option for patients with advanced RCC, conditional on pricing. Evidence is awaited regarding the efficacy of pazopanib compared to that of sunitinib in the first-line setting of advanced RCC (the COMPARZ trial). Based on the available evidence when designing the trial sunitinib is currently the only drug permitted for use in stages A/B of the STAR trial. When data becomes available from the COMPARZ trial, a decision will be made as to whether eligibility will be extended to patients treated with pazopanib. If pazopanib is permitted, then an additional minimisation factor will be required, as well as a recalculation of the QALY powering of the trial.

The STAR study will be an important trial. Chemotherapeutic agents have often necessitated restricted periods of treatment due to toxicity, but this is not necessarily true of novel targeted therapies. However, chronic minor side effects such as fatigue can have cumulative major effects on QoL, particularly over longer periods of time. Conflicting pressures exist when establishing a treatment duration, pharmaceutical companies have an interest in prolonged treatment durations, until disease progression or even beyond, but it is usually in the patient’s and healthcare provider’s best interest to use shorter durations of treatment, in terms of toxicity, QoL and cost effectiveness. If successful, the STAR trial design could be easily adapted for use with other systemic anti-cancer therapies in other disease sites. We predict that trial designs such as this will become increasingly utilised in the future, as they allow for incorporation of more complex treatment strategies alongside emphasising the importance of quality as well as quantity of life for patients.

### Endnotes

^a^TSF is defined as the time from randomisation until death; disease progression on sunitinib; disease progression in the DFIS arm during a planned treatment break assuming no further disease response or stabilisation on subsequent sunitinib occurs; participant requires use of a new systemic anti-cancer agent for RCC or significant clinical deterioration where deterioration is assumed to be due to renal cancer progression and not another comorbidity and deterioration sufficient to warrant cessation of sunitinib if on treatment or to preclude restarting sunitinib if on a treatment break on the DFIS arm, without it being clinicially appropriate to arrange radiological confirmation of the progression

^b^SPFI is defined as the sum of the intervals from the start of each treatment block with sunitinib, until radiological evidence of disease progression provided there has been some evidence of disease control prior to ongoing progression

## Abbreviations

5FU: 5 Fluorouracil; AE: Adverse Event; CCS: Conventional Continuation Strategy; CI: Confidence Invervals; CRC: Colorectal cancer; CRFs: Case Report Forms; CTRU: Clinical Trials Research Unit; DMEC: Data Monitoring and Ethics Committee; DFIS: Drug Free Interval Strategy; ECOG: Eastern Cooperative Oncology Group.; EQ-VAS: EuroQoL Visual Analogue Scale; EuroQoL: European Quality of Life [Group]; EVPI: Expected Value of Perfect Information; FA: Folinic Acid; FACT-G: Functional Assessment of Cancer Therapy – General; FKSI: Functional Assessment of Cancer Therapy-Kidney Symptom Index; GCP: Good Clinical Practice; HTA: Human Tissue Authority; IFNα: Interferon-α; IPT: Internal Project Team; ITT: Intention to treat; MHRA: Medicines and Healthcare products Regulatory Agency; MRU: Medical Resource Utilisation; NCRI: National Cancer Research Institute; NHS: National Health Service; NICE: National Institute for Health and Clinical Excellence; NIHR: National Institute of Health Research; OS: Overall Survival; PFS: Progression Free Survival; PSS: Personal Social Services; QALY: Quality Adjusted Life Year; QoL: Quality of Life; RCC: Renal Cell Cancer; RECIST: Response Evaluation Criteria in Solid Tumours; SAE: Serious Adverse Event; SAR: Serious Adverse Reaction; SD: Stable disease; Sd: Standard deviation; SOP: Standard Operating Procedure; SPC: Summary of Product Characteristics; SPFI: Summative Progression Free Inverval; SUSAR: Suspected Unexpected Serious Adverse Reaction; TKI: Tyrosine Kinase Inhibitor; TMG: Trial Management Group; TSC: Trial Steering Committee; TSF: Time to Strategy Failure.

## Competing interests

The authors declare that they have no competing interests.

## Authors’ contributions

Conception and Design of the STAR trial: JEB JMBB FJC TE WG JH CM PS TW, Protocol/Patient Information Sheet: JEB JMBB FJC TE WG JH PH CM CL BP IR PS ST TW, All authors have read and approved the final manuscript.

## Pre-publication history

The pre-publication history for this paper can be accessed here:

http://www.biomedcentral.com/1471-2407/12/598/prepub
